# *Parapoxvirus*-based therapy eliminates SARS-CoV-2-loaded fine aerosol and blocks viral transmission in hamster models

**DOI:** 10.3389/fmicb.2022.1086627

**Published:** 2022-12-01

**Authors:** Huan Cui, Kui Zhao, Cheng Zhang, Jing Lin, Shihui Sun, Qi Li, Le Du, Chunmao Zhang, Juxiang Liu, Feng Gao, Wenqi He, Yuwei Gao, Zhendong Guo, Jiyu Guan

**Affiliations:** ^1^Key Laboratory of Zoonosis, Ministry of Education, College of Veterinary Medicine, Jilin University, Changchun, China; ^2^Changchun Veterinary Research Institute, Chinese Academy of Agricultural Sciences, Changchun, China; ^3^College of Veterinary Medicine, Hebei Agricultural University, Baoding, Hebei, China

**Keywords:** SARS-CoV-2, aerosol transmission, immunomodulator, *Parapoxvirus*, hamster models

## Abstract

Currently, it is believed that severe acute respiratory syndrome coronavirus 2 (SARS-CoV-2) is an airborne virus, and virus-containing aerosol particles have been found concurrent with the onset of COVID-19, which may contribute to the noncontact transmission of SARS-CoV-2. Exploring agents to block SARS-CoV-2 transmission is of great importance to prevent the COVID-19 pandemic. In this study, we found that inactivated *Parapoxvirus ovis* (iORFV), a kind of immunomodulator, could compress the proportion of small particle aerosols exhaled by Syrian golden hamsters. Notably, the concentration of SARS-CoV-2 RNA-containing aerosol particles was significantly reduced by iORFV in the early stages after viral inoculation. Importantly, smaller aerosol particles (<4.7 μm) that carry infectious viruses were completely cleared by iORFV. Consistently, iORFV treatment completely blocked viral noncontact (aerosol) transmission. In summary, iORFV may become a repurposed agent for the prevention and control of COVID-19 by affecting viral aerosol exhalation and subsequent viral transmission.

## Introduction

It is currently believed that severe acute respiratory syndrome coronavirus 2 (SARS-CoV-2) can spread by airborne transmission, which is a dominant route (Greenhalgh et al., 2021). A supporting report showed that millions of SARS-CoV-2 can be exhaled from coronavirus disease 2019 (COVID-19) patients in the early stages (Ma et al., 2021). Syrian golden hamsters (hereafter “hamster”) are an ideal small animal model for studying SARS-CoV-2 including its pathogenicity and transmission, as well as vaccines, immunotherapies and antiviral drugs (Muñoz-Fontela et al., 2022). Animal experiments were set up to mimic SARS-CoV-2 transmission among humans, in which SARS-CoV-2-inoculated hamsters and ferrets displayed noncontact viral transmission to air-exposed animals (Richard et al., 2020; Sia et al., 2020). Importantly, direct evidence has confirmed that SARS-CoV-2-infected cynomolgus monkeys can exhale virus-laden aerosols, and the viruses could be released early after inoculation (Zhang et al., 2021). Additionally, SARS-CoV-2-inoculated hamsters have been found to produce infectious viruses in aerosol particles, which was accompanied by the onset of clinical signs (Hawks et al., 2021). Notably, SARS-CoV-2 can also undergo animal-to-human transmission. Up to 68% of workers in ferret farms were infected by SARS-CoV-2 strains with animal sequence signatures (Oude Munnink et al., 2021). Although no evidence shows cat-to-human transmission, airborne transmission between cats has been confirmed (Shi et al., 2020).

The inactivated *Parapoxvirus ovis* (iORFV) has been considered a nonspecific immunomodulating agent and has been studied in different species (Weber et al., 2013; Wang et al., 2019). iORFV treatment was effective in limiting the development of chronic viral diseases caused by hepatitis C virus (HCV) and hepatitis B virus (HBV; Weber et al., 2003). In addition, treatment with iORFV protected mice from the lethal HSV-1 challenge (Weber et al., 2003). ORFV-based immunomodulation could also reduce the incidence of acute respiratory disease in animals (Castrucci et al., 1996; Ziebell et al., 1997). Of note, iORFV treatment also displays therapeutic potential in antifibrotic activity in animal models of liver fibrosis (Nowatzky et al., 2013). The recruitment of immune cells and the upregulation of associated cytokines such as interferon-α, interleukin-1β and granulocyte-macrophage colony-stimulating factor are involved in the iORFV-induced response (Paulsen et al., 2013; Anziliero et al., 2014).

Here, we repurpose this nonspecific agent to explore its capacity to limit SARS-CoV-2 transmission. We collected exhaled aerosol particles from virus-inoculated hamsters and examined the size distribution of aerosols, virus-containing aerosols and viral transmission between animals.

## Materials and methods

### Viruses and cells

SARS-CoV-2 BetaCoV/Beijing/IME-BJ05-2020 (Biological Sample Library: SAMC138020) was propagated and titrated in African green monkey kidney epithelial cells (Vero E6; CRL1586, ATCC, United States). The cell line was maintained in high-glucose Dulbecco’s modified Eagle’s medium (DMEM; Invitrogen, Carlsbad, CA, United States) supplemented with 10% fetal bovine serum (FBS; Gibco, Auckland, New Zealand), containing 100 U/ml penicillin, and 100 μg/ml streptomycin. The cells were incubated at 37°C in a humidified incubator containing 5% CO_2_. Viral titers were determined using a standard tissue culture infective dose 50% (TCID_50_) assay.

### Preparation of iORFV

The ORFV strain OV-SY17 (GenBank accession number MG712417) was propagated in primary OFTu cells. OFTu cells were maintained in DMEM supplemented with 10% FBS, 100 U/ml penicillin, and 100 μg/ml streptomycin. The viruses were harvested when 80%–90% of the cytopathic effect emerged. The cytopathic cells were collected and subjected to three cycles of repeated freeze–thaw cycles, which were followed by low-speed centrifugation to remove cell debris. The viruses were then purified through sucrose gradient ultracentrifugation. The concentrated virus solution was quantified after dilution and presented as TCID_50_/mL. ORFV was then inactivated with beta-propiolactone (1:4,000–1:2,000) at 4°C for 24 h to inactivate ORFV and then at 37°C for 2 h to inactivate beta-propiolactone. The inactivated virus particles were then stored at −80°C until use.

### Collection of exhaled viral aerosols from hamsters

Six hamsters were randomly divided into two groups of 3 hamsters, a positive control group and a treatment group. The hamsters in both groups were inoculated with 10^5.0^ TCID_50_ of SARS-CoV-2. The hamsters were treated with iORFV (10^6.0^ TCID_50_) or vehicle separately for five consecutive days. Exhaled aerosols were collected from our published studies (Guo Z. et al., 2022). An Andersen-6 sampler (TE-20-800, TISCH, Cleves, OH, United States) was used to collect exhaled aerosol samples from different groups of hamsters at a flow rate of 28.3 L/min for 1 h at 2, 3, 5, and 7 days post-infection (dpi). The Andersen-6 sampler was fractionated based on the aerodynamic particle diameters as follows: 0.65–1.1 μm, 1.1–2.1 μm, 2.1–3.3 μm, 3.3–4.7 μm, 4.7–7.0 μm and ≥7.0 μm. The sampler was sufficiently disinfected with 75% alcohol and dried before each sampling. Exhaled aerosol samples of hamsters were collected using presterilized gelatin filters (Sartorius, Germany), and three independent biological replicates were performed for each group at each time point. Each filter membrane was cut into two equal pieces after aerosol sampling. One piece was used for RNA extraction and nucleic acid detection, and the other was seeded directly into Vero E6 cells to determine the presence of infectious viruses in aerosols.

### SARS-CoV-2 transmission studies in hamsters

The studies were carried out in an ABSL3 facility at Changchun Veterinary Research Institute. All of the animal studies were performed in strict accordance with the guidelines set by the Chinese Regulations of Laboratory Animals and Laboratory Animal Requirements regarding Environment and Housing Facilities. All animals used in this study were chosen randomly. Six-week-old male hamsters (Merial Vital Laboratory Animal Technology Company) were used in this study. In the transmission studies, three hamsters per group were intranasally (i.n.) inoculated with 100 μl of the test viruses at 10^5.0^ TCID_50_ and housed in a cage placed inside an isolator. The next day, these three hamsters were housed in a wire-frame cage adjacent to the three SARS-CoV-2-inoculated hamsters to study aerosol transmission. The distance between the SARS-CoV-2-inoculated and aerosol-contact hamster cages was 5 cm. To monitor virus shedding, nasal washes were collected and titrated from all animals at the indicated time points.

## Results and discussion

**Figure 1:**
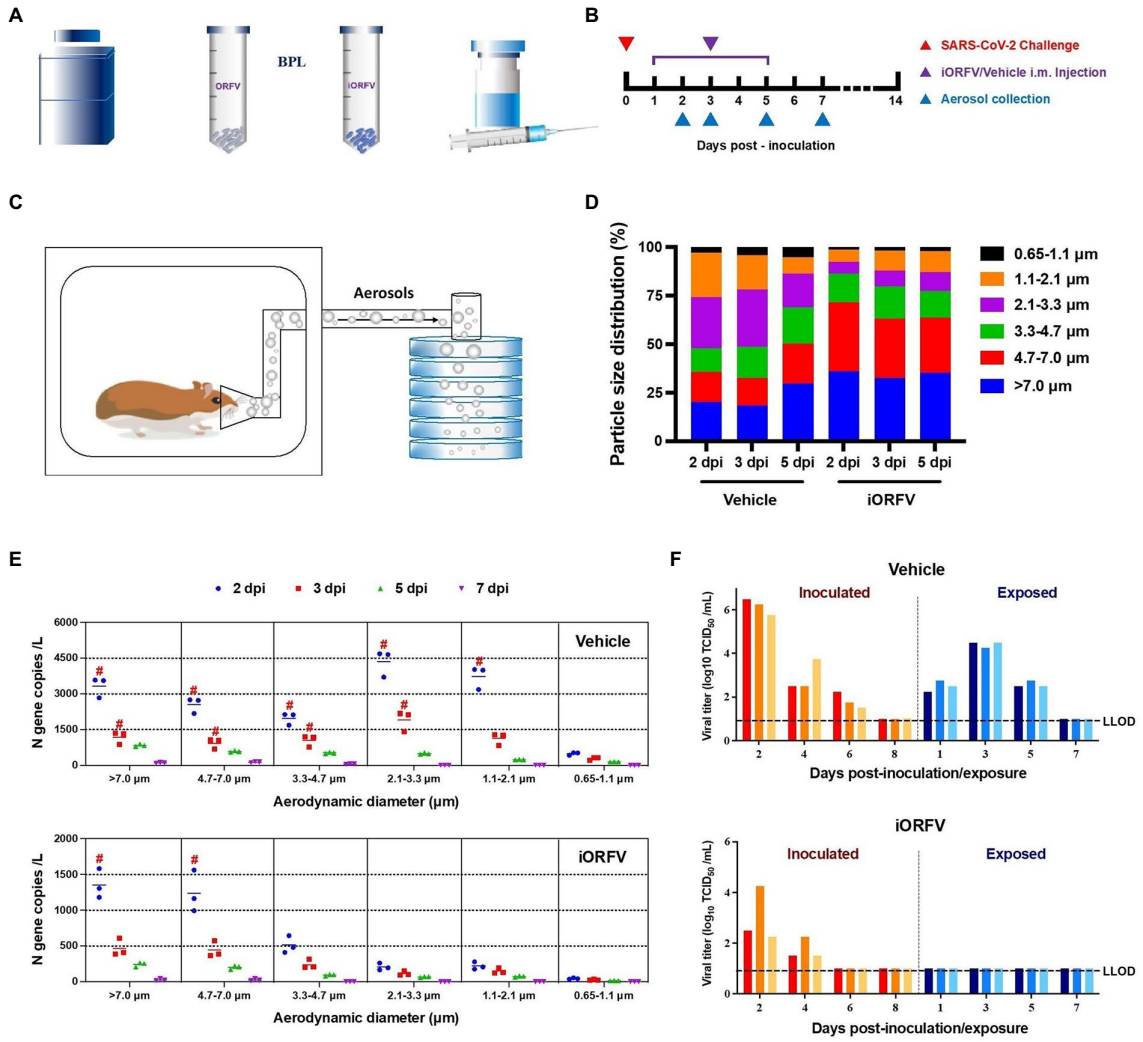
The effect of iORFV on viral aerosol distribution and transmission in hamsters. **(A)** Schematic map of the preparation of inactivated ORFV. **(B)** Schematic design of iORFV i.m. treatment for 5 consecutive days after SARS-CoV-2 challenge in hamsters. **(C)** Hamster-exhaled aerosols were collected and analyzed in a 6-stage Andersen sampler. **(D)** The particle size distributions of aerosol exhaled in the two groups of hamsters at 2, 3, 5 dpi. **(E)** Quantity and size distribution of exhaled viral particle-loaded aerosols in SARS-CoV-2-inoculated hamsters at 2, 3, 5 and 7 dpi. The red # indicates that infectious virus was detected in the aerosol sample. **(F)** SARS-CoV-2 was transmitted from donor to recipient hamsters (aerosol-exposed hamsters) under treatment with either vehicle or iORFV. Nasal washes were collected and analyzed from both donor and recipient hamsters. The lower limit of detection (LLOD) for viral titers is indicated with a black dotted line.

iORFV is a nonspecific immunomodulator that can inhibit virus-related diseases and has been studied for more than three decades in different species (Friebe et al., 2004; Weber et al., 2013; Wang et al., 2019). iORFV treatment is effective in limiting the development of chronic viral diseases caused by hepatitis C virus (HCV) and hepatitis B virus (HBV; Weber et al., 2003; Paulsen et al., 2013). iORFV immunomodulation could also reduce the incidence of acute respiratory disease in animals (Castrucci et al., 1996; Ziebell et al., 1997). Furthermore, iORFV treatment has been shown to have antifibrotic activity in animal models of liver fibrosis (Nowatzky et al., 2013). Here, we inactivated ORFV with beta-propiolactone (BPL) and identified its morphology (Figure 1A; Supplementary Figure 1). The hamsters were treated (intramuscular injection, i.m.) with iORFV for five continuous days after i.n. SARS-CoV-2 inoculation (Figure 1B). Hamster-exhaled aerosol samples were collected and measured by a 6-stage Andersen sampler (Figure 1C). Fine particles (<4.7 μm) from hamsters inoculated with SARS-CoV-2 maintained dominance, especially in the early stages (2 and 3 dpi), and the ratio accounted for more than 60%. In contrast, the ratio dropped to ~30% under iORFV treatment (Figure 1D). Notably, iORFV compressed the proportion of aerosol particles in the ranges of 0.65–1.1 μm, 1.1–2.1 μm, and 2.1–3.3 μm (Figure 1D), indicating that iORFV significantly altered the size distribution of exhaled aerosols.

In general, the total quantity of viral aerosols exhaled by iORFV-treated hamsters was significantly lower than that exhaled by control hamsters (only SARS-CoV-2-inoculated) at all time points (2, 3, 5 and 7 dpi; Supplementary Figure 2). In the early stage (2 dpi), the concentration of viral aerosol particles exhaled by the control group was 16,512.66 ± 1574.94 copies per liter of air. In contrast, the concentration of viral aerosol particles exhaled by the iORFV-treated hamsters was 3481.46 ± 670.25 copies per liter of air, and the concentration of viral aerosol particles exhaled by the iORFV-treated hamsters was only one-fifth that of the control group. Additionally, infectious viruses were detected only at 2 dpi in iORFV-treated animals (Supplementary Figure 2). To illustrate a more detailed size distribution of exhaled viral particles, we examined viral RNA copies in aerosol particles of different sizes. In the early stages (2 and 3 dpi) after SARS-CoV-2 inoculation, viral RNA from exhaled aerosol particles of all sizes was significantly reduced under iORFV treatment (Figure 1E; Supplementary Table 1). Of note, infectious viruses were found in both larger (>7.0 μm and 4.7–7.0 μm) and fine (<4.7 μm) aerosol particles from control hamsters at 2 and 3 dpi, while they were detected only in larger (>7.0 and 4.7–7.0 μm) particles from iORFV-treated hamsters at 2 dpi (Figure 1E). The above results demonstrate that iORFV limits the exhalation of SARS-CoV-2-containing aerosols, especially fine particles, indicating its influence on viral transmission.

To prove the above hypothesis, recipient hamsters were exposed to SARS-CoV-2-inoculated hamsters (donors) in two adjacent cages, and the therapeutic and viral challenge strategies are shown in the schematic map (Supplementary Figure 3). Three days post-exposure (dpe), the viruses were detected in the nasal washes of recipient hamsters, and the viral load peaked (Figure 1F), indicating that viral transmission from donor to recipient animals occurred through aerosols. After the performance of iORFV therapy, viral aerosol transmission from the donors to recipients was completely blocked, as infectious SARS-CoV-2 was undetectable in nasal washes of recipient hamsters at any time point (Figure 1F). Additionally, viral clearance was also accelerated in the donors at 6 dpi compared with that in their counterparts in the control group (Figure 1F). These data supported that iORFV therapy was able to block viral aerosol transmission and accelerate viral clearance in cases of infection.

In the present study, we found that *parapoxvirus*-based therapy could change the size distribution of exhaled aerosols and reduce SARS-CoV-2-containing aerosols. This strategy can further block viral aerosol transmission. To our knowledge, this is the first report to show that a repurposed agent can block SARS-CoV-2 transmission by affecting the exhaled viral aerosols.

SARS-CoV-2 transmission in community and healthcare settings is still a major problem that people are facing. Viral aerosols are considered the key factor for transmission. For example, exhaled fine viral aerosols in the community remain in suspension for a very long time, which increases the risk of more infections. Although air contamination of SARS-CoV-2 is controlled in many healthcare facilities because of the ventilation system, an infection may still occur during unnoticed aerosol-generating procedures (Romano-Bertrand et al., 2021). Thus, exploring agents that could block viral aerosol exhalation is a constructive strategy to reduce the COVID-19 epidemic.

Hamster is naturally susceptible to SARS-CoV-2 infection and shows mild clinical symptoms of COVID-19, which is a nice model to study the noncontact transmission of SARS-CoV-2 (Sia et al., 2020). Direct evidence shows that aerosol particles from SARS-CoV-2-inoculated hamsters contain infectious SARS-CoV-2 (Hawks et al., 2021), indicating that hamsters are a suitable platform for exploring agents that block SARS-CoV-2 transmission.

Although infectious viruses were detected in the iORFV-treated group at 2 dpi (Figure 1E), iORFV was still effective in blocking the aerosol transmission of SARS-CoV-2 in all stages (Figure 1F). The following reasons may help to explain the above phenomenon: First, it was reported that the concentration of exhaled virus aerosols determines the transmissibility of the virus and the infection risk of susceptible people (Guo W. et al., 2022). One report has shown that COVID-19 patients exhale millions of SARS-CoV-2 particles per hour, indicating the important role aerosol concentration may play in virus transmission (Ma et al., 2021). Thus, the significantly reduced total viral aerosol concentrations in the treatment group may be one of the factors (Supplementary Figure 2). Second, viral particles smaller than 4.7 μm stay in the air longer, spread more widely and play a more important role in aerosol transmission (Tang and Li, 2007). Here, infectious virus particles smaller than 4.7 μm were absent at any time point in aerosols from iORFV-treated animals (Figure 1E), which may partially explain the blockade of viral transmission.

Regarding the mechanisms of iORFV-induced viral aerosol reduction, there are two possibilities. First, iORFV therapy may affect the replication of SARS-CoV-2 in the upper respiratory tract, as ACE2 receptors are highly expressed in the upper respiratory tract, which may affect viral shedding in the form of aerosols (Romano-Bertrand et al., 2021). Second, the lower respiratory tract has been proven to exhale the smallest respiratory particles (Romano-Bertrand et al., 2021). Thus, we hypothesize that iORFV therapy may also alleviate SARS-CoV-2 loads in the lower respiratory tract and lead to the reduction of fine viral aerosols. Therefore, the target of the iORFV agent may be in the respiratory tract, and related mechanisms still need to be explored.

## Conclusion

In summary, iORFV may become a repurposed agent for the prevention and control of COVID-19 by affecting viral aerosol exhalation and subsequent viral transmission.

## Data availability statement

The original contributions presented in the study are included in the article/Supplementary material, further inquiries can be directed to the corresponding authors.

## Ethics statement

The animal study was reviewed and approved by the relevant regulatory agency of the Animal Ethical Committee of Changchun Veterinary Research Institute.

## Author contributions

JG, ZG, and YG designed this study. HC, KZ, CZ, and JL conducted the experiments. SS, QL, LD, CMZ, JXL, and FG analyzed and visualized the data. JG, CZ, and HC wrote the manuscript. WH, ZG, and YG revised the manuscript. All authors contributed to the article and approved the submitted version.

## Funding

This research was supported by grants from the National Major Research & Development Program (2020YFC0840800), a training plan for outstanding young teachers of Jilin University (grant: 419080520416) and the Science and Technology Innovation Special Fund Project of Jilin Province (20200402052NC).

## Conflict of interest

The authors declare that the research was conducted in the absence of any commercial or financial relationships that could be construed as a potential conflict of interest.

## Publisher’s note

All claims expressed in this article are solely those of the authors and do not necessarily represent those of their affiliated organizations, or those of the publisher, the editors and the reviewers. Any product that may be evaluated in this article, or claim that may be made by its manufacturer, is not guaranteed or endorsed by the publisher.
