# Supplementary material for: Parapoxvirus-based therapy eliminates SARS-CoV-2-loaded fine aerosol and blocks viral transmission in hamster models
Source: Front Microbiol. 2022 Dec 1;13:1086627. doi: 10.3389/fmicb.2022.1086627 (PMC9751573; doi:10.3389/fmicb.2022.1086627)
Supplement: Supplementary file 1 [file Data_Sheet_1.docx]

#
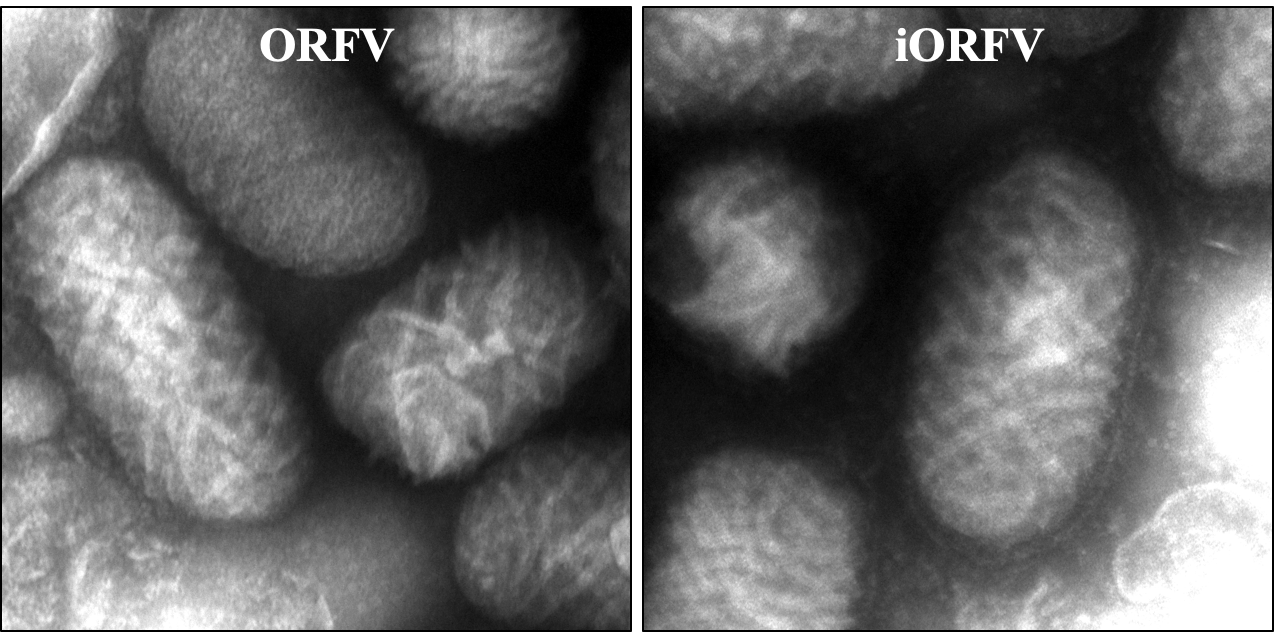


**Supplementary Figure 1.** The morphology of live and inactivated ORFV.

**Supplementary Figure 2.** Total concentration of exhaled viral aerosols from SARS-CoV-2-inoculated hamsters at 2, 3, 5, and 7dpi. **p < 0.01 and ***p < 0.001. The red # indicates that infectious virus was detected in the aerosol sample.


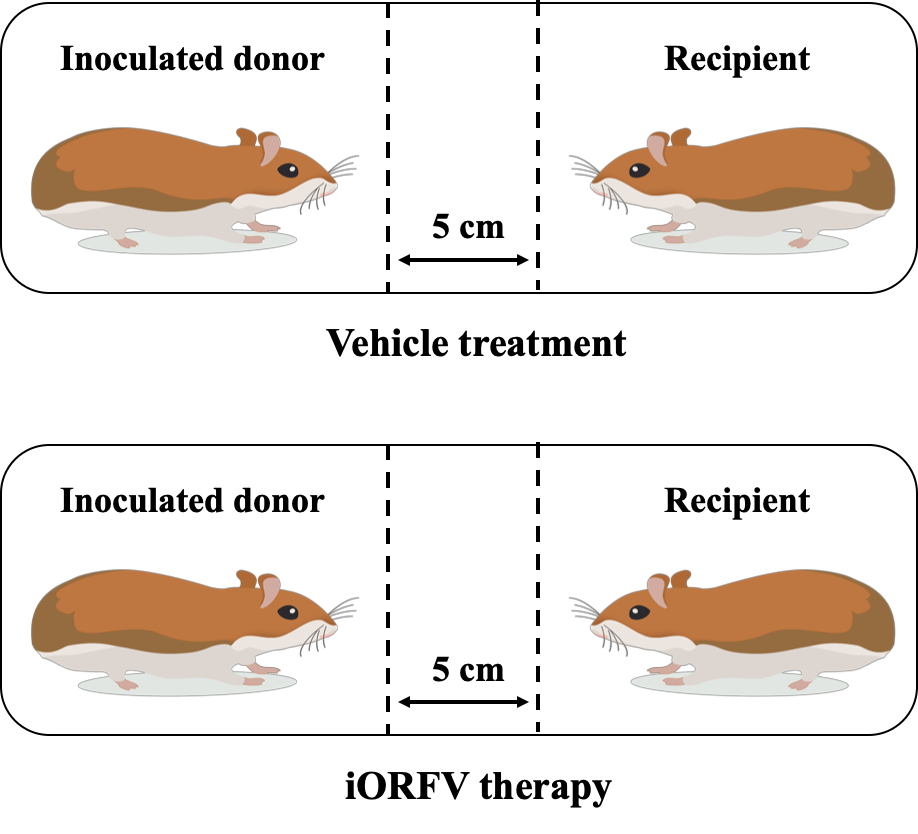


**Supplementary Figure 3.** Experimental SARS-CoV-2 transmission was carried out by aerosol contact. Vehicle- or iORFV-treated hamsters were challenged with SARS-CoV-2 and then housed in two adjacent cages.

**Supplementary Table 1.** The quantity and size distribution of viral RNA-loaded aerosol particles exhaled by SARS-CoV-2-inoculated hamsters at 2, 3, 5, and 7dpi.

| **Treatment** | **Size** | **2 dpi** | **3 dpi** | **5 dpi** | **7 dpi** |
| --- | --- | --- | --- | --- | --- |
| Vehicle | >7.0 μm | 3323±425 | 1179±265 | 839±45 | 101±17 |
| iORFV | >7.0 μm | 1357±206 | 467±121 | 244±30 | 25±15 |
| Vehicle | 4.7-7.0 μm | 2550±326 | 925±208 | 579±31 | 131±23 |
| iORFV | 4.7-7.0 μm | 1240±292 | 441±114 | 199±24 | 23±13 |
| Vehicle | 3.3-4.7 μm | 1976±253 | 1049±236 | 523±28 | 49±8 |
| iORFV | 3.3-4.7 μm | 512±120 | 240±62 | 95±11 | 0 |
| Vehicle | 2.1-3.3 μm | 4342±556 | 1902±428 | 489±26 | 0 |
| iORFV | 2.1-3.3 μm | 207±49 | 114±29 | 67±8 | 0 |
| Vehicle | 1.1-2.1 μm | 3733±478 | 1126±253 | 239±12 | 0 |
| iORFV | 1.1-2.1 μm | 220±51 | 149±38 | 73±9 | 0 |
| Vehicle | 0.65-1.1 μm | 485±62 | 279±62 | 146±7 | 0 |
| iORFV | 0.65-1.1 μm | 43±10 | 26±7 | 15±1 | 0 |
